# The Role of Exosomes in the Progression and Therapeutic Resistance of Hematological Malignancies

**DOI:** 10.3389/fonc.2022.887518

**Published:** 2022-05-26

**Authors:** Haobing Wang, Yong You, Xiaojian Zhu

**Affiliations:** ^1^ Institute of Hematology, Union Hospital, Tongji Medical College, Huazhong University of Science and Technology, Wuhan, China; ^2^ Department of Hematology, Tongji Hospital, Tongji Medical College, Huazhong University of Science and Technology, Wuhan, China

**Keywords:** exosome, hematological malignancies, therapy resistance, immunosuppression, tumor progression

## Abstract

Exosomes are membrane limited structures which derive from cell membranes and cytoplasm. When released into extracellular space, they circulate through the extracellular fluid, including the peripheral blood and tissue fluid. Exosomes surface molecules mediate their targeting to specific recipient cells and deliver their contents to recipient cells by receptor-ligand interaction and/or phagocytosis and/or endocytosis or direct fusion with cell membrane. Exosomes contain many functional molecules, including nucleic acids (DNAs, mRNAs, non-coding RNAs), proteins (transcription factors, enzymes), and lipids which have biological activity. By passing these cargos, exosomes can transfer information between cells. In this way, exosomes are extensively involved in physiological and pathological processes, such as angiogenesis, matrix reprogramming, coagulation, tumor progression. In recent years, researcher have found that exosomes from malignant tumors can mediate information exchange between tumor cells or between tumor cells and non-tumor cells, thereby promoting tumor survival, progression, and resistance to therapy. In this review, we discuss the pro-tumor and anti-therapeutic effects of exosomes in hematological malignancies, hoping to contribute to the early conquest of hematological malignancy.

## Introduction

**Figure 1:**
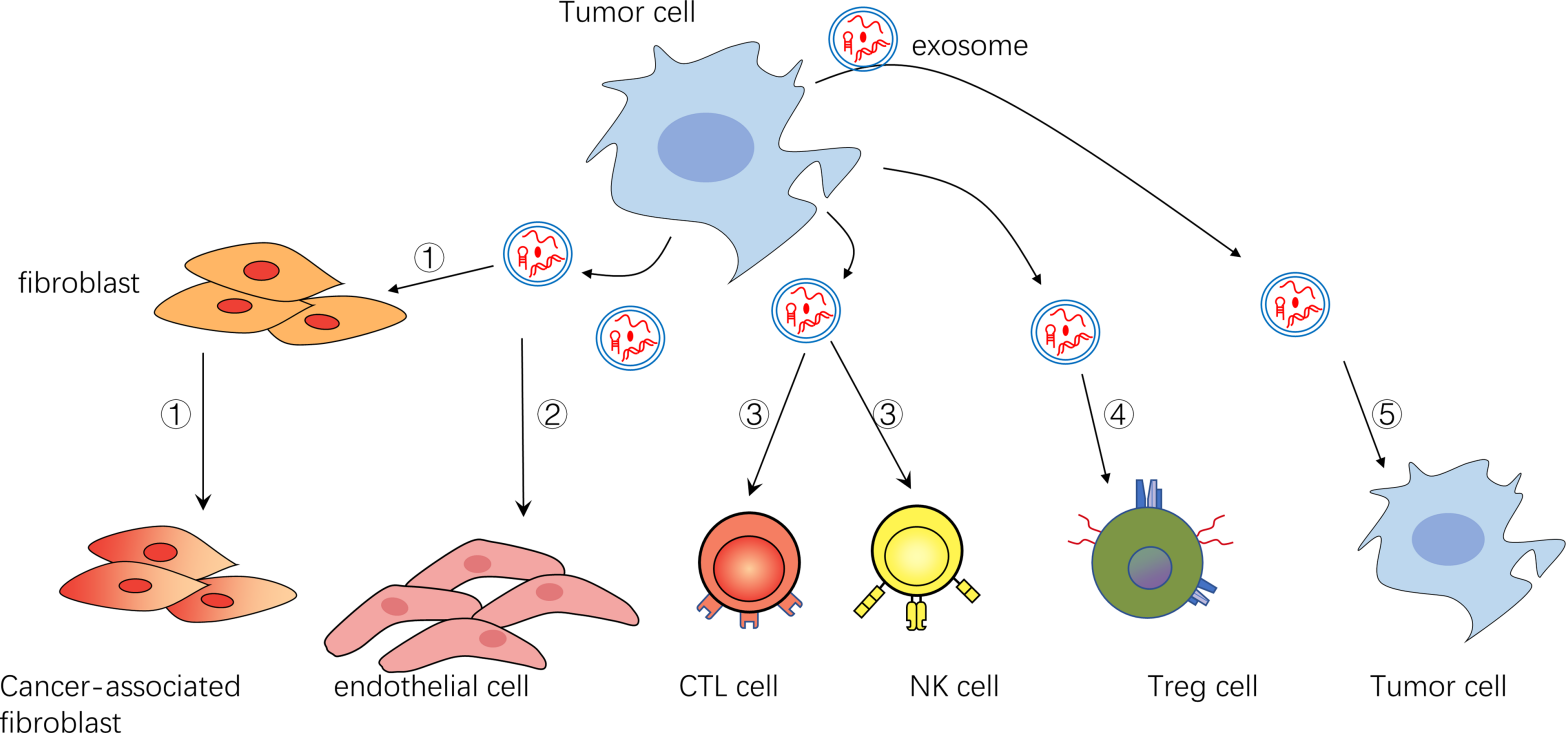
**①**Exosomes can transform fibroblasts into tumor-associated fibroblasts **②**Exosomes can promote endothelial cell growth and vascular formation **③**Exosomes can inhibit NK cells and CTL cells **④**Exosomes can activate immunomodulatory cells such as Treg cells **⑤**Exosomes can act on themselves or their surrounding tumor cells to promote their survival and inhibit their apoptosis.

**Figure 2:**
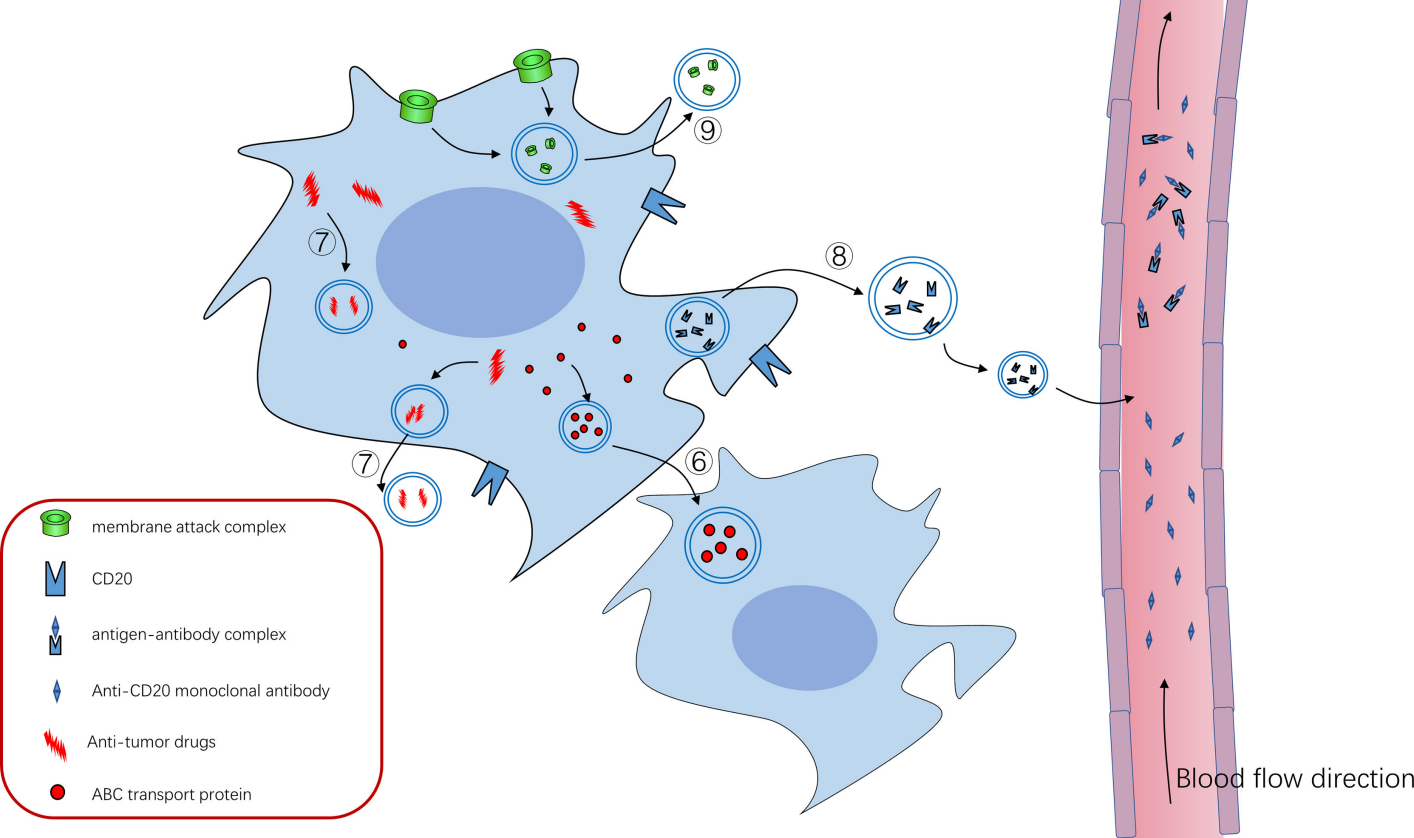
**⑥**ABC transporter can mediate the formation of exosomes and drug resistance of tumor cells, and ABC transporter can be “shared” among tumor cells through exosomes to promote tumor progression **⑦**Tumor cells can use exosomes to isolate or excrete intracellular antitumor drugs to achieve therapeutic resistance **⑧**CD20 exists on the cell membrane of B cells, and anti-CD20 Rituximab can bind to CD20 to kill tumor cells. Tumor cells use exosomes to package some CD20 as decoy to release cells, which combine with anti-CD20 Rituximab to reduce their bioavailability and protect tumor cells from killing effects **⑨**Tumor cells use exosomes to expel the membrane attack complex (MAC) inserted into the cell membrane to the outside of the cell, reducing its killing effect.

Exosomes are particles (30-150 nm in diameter) secreted by cells that are wrapped in lipid bilayer and contain various cargo on and within the membrane molecule surface. And because they have no functional nucleus, they can’t replicate themselves (1). Since Johnstone et al. discovered them in 1983 during transferrin receptor recycling in maturation of sheep reticulocytes (2) and subsequently named them exosomes (3), exosomes have been extensively studied about their biogenesis, their role in tumor progression and therapy resistance. Exosomes (sometimes only identified as small extracellular vesicles) can also transmit information through the bioactive nucleic acids (DNA, mRNA, miRNAs, CircRNAs), proteins (transcription factors, enzymes), and lipids that they contain (4–6). In addition, unlike conventional information molecules, exosomes have specific integrin profiles on their membranes, enabling them to target specific tissues and cells (7). After reaching their target tissue or target cell, by receptor-ligand interaction and/or phagocytosis and/or endocytosis and/or direct fusion with cell membrane, exosome cargos can directly regulate the function of recipient cells or alter their gene expression. So far, it has been found that the amount and contents of exosomes or other extracellular vesicles in tumor patients are quite different from those in normal patients (8, 9). In addition, the exosomes of tumor patients are different before and after treatment. Researchers found that, in both adult and pediatric acute myelogenous leukemia (AML), the amount of double-stranded DNA in the extracellular vesicles (EVs), which conclude exosomes, was consistent with the disease state. The amount of DNA in EVs in plasma decreases after treatment and increases after relapse (10–12). However, there are also some data suggesting fluctuations in plasma EVs levels in AML patients, with an initial sharp decline and a gradual increase during treatment. Whether this is due to increased resistance of tumor cells to treatment is unclear (11). In conclusion, these results suggest that exosomes are involved in the progression of tumor diseases. And many more specific studies have shown that exosomes promote tumor cell survival and disease progression in the following ways. 1. Promoting angiogenesis to supply nutrition, inhibiting apoptosis, and delivering pro-survival cytokines. 2. Promoting matrix reprogramming into a pro-tumor niche. 3. Inhibiting anti-tumor immune cells such as NK cells, CTL cells, and promoting the function of immunomodulatory cells. 4. Promoting resistance therapy by efflux drugs, and directly sequester drugs. The role of exosomes in hematological malignancies will be described in detail in the following sections. In addition, there have been reviews on topics similar to this review. But there is still a certain novelty in our paper (13–15). 1. Most of the reviews on similar topics describe that EVs secretion by tumor cells affects stromal cells and immune cells. But our manuscript also shows that tumor cells support each other by exchanging exosomes. 2. The evidence for our opinion mainly comes from the research of hematological tumors, and the evidence from solid tumors is rarely used, which makes this paper more reliable for researchers in the field of hematologic tumor. The diseases involved in this paper are briefly introduced in **Table 1**. All the molecules covered in this paper are shown in **Table 2**. In addition, for the convenience of readers, we added the mechanism diagram of exosome's influence on tumors. These two figures are presented in the paper in the form of **Figure 1** and **Figure 2**.

**Table 1:** Features of various hematological malignancies present in the manuscript.

Disease	Features	Reference
AML	Malignant clonal proliferation of hematopoietic stem cells progenitor cells that should have differentiated into neutrophils, eosinophils, basophils, and monocytes. This occurs in bone marrow, blood and other tissues and inhibits normal hematopoiesis.	(16–23)
ALL	Abnormal proliferation of lymphocyte progenitors leads to excess lymphoblasts in bone marrow and blood and inhibits normal hematopoiesis.	(24, 25)
CML	the myeloid primitive cells proliferate maliciously as a result of the Ph chromosome, the differentiation of malignant cells stagnated at a later stage, and malignant cells are more mature than acute leukemia.	(26–33)
CLL	Slow progressing disease caused by malignant proliferation of mature B lymphocytes. A large number of clonal B lymphocytes were found in bone marrow, peripheral blood and lymphoid tissues.	(34, 35)
MM	Abnormal proliferation of clonal plasma cells in the bone marrow and in most cases there is secretion of monoclonal immunoglobulin or its fragments.	(36–40)
lymphoma	During lymphocyte proliferation and differentiation process, malignant transformation at a specific stage. Then the malignant cells proliferate to form the tumor of the immune system.	(41–45)

**Table 2:** Exosome cargos and their function in tumor progression.

Molecule	Disease	Effect	Reference
**RNA**			
miR-135b	MM	suppressed FIH-1, enhance HIF-1 bioactivity, promote angiogenesis	(36)
miR-92a	CML	promoted the formation of vascular tubular structures	(26)
miR-365	CML	inhibited the expression of Caspase3 and apoptosis of tumor cells	(28)
hTERT mRNA	CML	promoted fibroblasts to transform into CAF that promote leukemia cells survival	(29)
IGF-IR mRNA MMP9 mRNA	AML	promoted the proliferation of stromal cells and promoted stromal cells to secrete growth factors	(17)
BCR-ABL1 mRNA	CML	promoted BM-MSC to transform into a pro-tumor phenotype	(32)
Y RNA hY4	CLL	transformed monocytes into an immunosuppressive phenotype	(35)
miR-20a miR-196	AML	expelled specific RNAs that not conducive to tumor survival out of cells	(23)
**Protein**			
TGF-β	CML	promoted tumor cells survival and proliferation	(27)
IL-6, CCL2, Fibronectin	MM	induced the survival, proliferation, and migration of tumor cells	(37)
Amphiregulin	CML	activated EGFR signaling of stromal cells, increased expression of IL-8	(30, 31)
Heparinase	MM	promoted macrophages to secrete TNF-α	(39)
LMP1	Burkitt lymphoma	induced primary B cells to proliferate and exhibit a malignant phenotype	(41)
IL-10, TGF-β	AML	promoted CD4^+^CD25^-^ T cells to transform into CD4^+^CD25^+^Foxp3^+^T cells, namely Treg cells	(20)
FasL, PD-L1, TGF-β	AML	inhibited anti-tumor immune cells	(18, 19, 21)
CK2	lymphoma	phosphorylated C9 to protect tumor cells	(43)
MICA/B	leukemia/ lymphoma	reduced the cytotoxic effect of NK cells	(42)
MAC	CML	expelled the cancer-killing MAC from tumor cells	(33)
P-gp	ALL	trapped anti-tumor drugs in membrane structures	(24, 25)
ABCA3	AML	trapped anti-tumor drugs in membrane structures	(22)
MRP1	APL	transferred drug-resistant proteins between tumor cells	(23, 46, 47)
CD20	lymphoma	combined with rituximab, reduced its bioavailability	(45)
**Others**			
Doxorubicin, pixantrone	lymphoma	expelled the chemotherapeutic drugs out of cells	(44)
Uncertain	CLL	promoted endothelial cell migration and angiogenesis;promoted proliferation and secretion of inflammatory cytokines of stromal cells	(34)
Uncertain	MM	activated pro-survival signaling pathways, including JNK, p38, p53, Akt pathway, increased the expression of antiapoptotic protein Bcl-2	(38)
Uncertain	AML	up-regulated the expression of anti-apoptotic protein Bcl-2	(16)
Uncertain	AML	down-regulated the expression of JAK3 (Janus kinase 3) and CD3ζ in activated T cells, thereby inhibiting the function of CD8^+^T cell	(20)
Uncertain	MM	regulated STAT3, promoted the growth of MDSCs and enhanced its immunosuppressive activity	(40)

The “uncertain” in the table means that the researchers found that the corresponding exosome do affect tumor cells, but it is not clear which molecule is responsible.

## Exosomes Have the Function of Promoting the Survival and Progression of Tumor Cells

### Promoting Angiogenesis

Tumor angiogenesis is closely related to disease progression. Tumor cells secrete angiogenic factors such as vascular endothelial growth factor (VEGF), fibroblast growth factor (FGF), transforming growth factor-β(TGF-β), hypoxia inducible factor-1 (HIF-1) and Interleukin-8 (IL-8), which promote the proliferation and growth of endothelial cells, and the new born blood vessels provide nutrition and oxygen for tumor tissue (36, 48). This exists in the whole process of tumor disease progression. In the field of hematological tumors, researchers have found that exosomes (or small extracellular vesicles) secreted by a variety of hematological malignancies can promote angiogenesis in the bone marrow (26). In malignant myeloma, the proliferation of plasma cells leads to hypoxia. In this case, tumor cells secrete exosomes that contain miR-135b. And then miR-135b exert its function to suppress its molecule target factor-inhibiting hypoxia-inducible factor 1 (FIH-1) after it is transported to the target cell vascular endothelial cell. In this way, exosomes secreted by multiple myeloma cells can enhance HIF-1 bioactivity, thereby promoting angiogenesis (34). In chronic myelogenous leukemia (CML), exosomes derived from K562 cells line are internalized by human umbilical endothelial cells (HUVECs), activating the Src signaling pathway. These endothelial cells have an increased tendency to form tubular structures, ultimately promoting angiogenesis (49). In another study, exosomes derived from K562 cells containing miR-92a have the same effect as to promote the formation of vascular tubular structures (50). Besides, exosomes derived from chronic lymphocytic leukemia (CLL) promote endothelial cell migration and angiogenesis *in vitro* and *in vivo* (51, 52).

### Activating Pro-Survival Signal Pathway

Through paracrine and/or autocrine, exosomes derived from tumor cells or stromal cells deliver growth factors, extracellular enzymes, or other factors necessary for tumor growth to promote tumor progression, such as transforming growth factor-β1 (TGF-β1), Caveolin-1, and Wnt (27). These factors can promote the survival of tumor cells and increase their invasiveness and migration ability (37, 53). For example, CML cell-derived exosomes contain TGF-β1, which interacts with TGF-β1 receptors on the surface of tumor cells to promote cells survival and proliferation (38). Treatment with TGF-β1 inhibitors significantly reduced exosome-stimulated cell proliferation and colony formation (54). *In vivo* multiple myeloma mouse model, bone marrow stromal cell (BMSC)-derived exosomes contain oncoproteins, cytokines, adhesion molecules, such as IL-6, CCL2, and fibronectin, which can induce the survival, proliferation, and migration of multiple myeloma cells. Whereas normal BMSC-derived exosomes significantly reduced myeloma cell proliferation (16). In addition, researchers have found that exosomes obtained from myeloma patients’ BMSC, when taken by tumor cells, can affect several signaling pathways related to cell proliferation, differentiation, and apoptosis, including c-Jun N-terminal kinase (JNK), p38, p53, and Akt pathway (28). In diffuse large B-cell lymphoma, Koch et al. found that Wnt signaling activated by exosomes can induce non-side population cells to exhibit a side population (SP) phenotype. This side population of cells has characteristics like those of tumor stem cells, which involve in the occurrence, progression, metastasis, and recurrence of tumor diseases (55).

### Suppressing Apoptosis of Tumor Cells

When the immune surveillance function of the body is normal, the malignant cells will initiate programmed cell death, avoiding the progression into malignant tumors. The apoptotic ability of cells depends to a large extent on the relative expression of anti-apoptotic protein (namely Bcl-2, Bcl-xl etc.) and pro-apoptotic protein (namely Bax, Caspases etc.). When the anti-apoptotic protein system gains the advantage, tumor cells exhibit an anti-apoptotic phenotype, these cells eventually develop into malignant tumors. In hematological malignancies, exosomes secreted by tumor cells or stromal cells can mediate the anti-apoptotic process. Researcher have found that, in the murine multiple myeloma model, tumor cells treated with exosomes derived from BMSCs show a unique protein expression profile. Antiapoptotic protein Bcl-2 increases whereas proapoptotic protein Bax don’t change. Besides, Full-length caspase-9 increases, cleaved caspase-9 and caspase-3 reduce, which means reduced pro-apoptotic activity of the caspase (28). In another research, exosomes (or small extracellular vesicles) derived from apoptotic-resistant AML cells containing proteins with apoptotic regulatory functions. When apoptotic-sensitive AML cells are co-cultured with exosomes of apoptotic-resistant AML cells, the expression of anti-apoptotic protein Bcl-2 will up-regulate. Accordingly, such AML cells exhibit anti-apoptotic, growth-promoting properties (56).

Researchers found that exosomes derived from imatinib-resistant CML cells contain high level of miR-365, and they can be internalized into drug-sensitive CML cells and imbibe resistance characteristics. In addition, imatinib-sensitive CML cells transfected with pre-miR-365 showed lower chemotherapy sensitivity and apoptosis rate, which also confirmed the anti-apoptosis function of miR-365. Further studies show that miR-365 induce drug resistance by inhibiting the expression of pro-apoptotic protein (Caspase3) in CML cells. In conclusion, these studies suggests that exosomes (or other extracellular vesicles) can mediate drug-resistant horizontal transfer between CML cells (57, 58).

### Promoting the Transformation of Stromal Cells Into Pro-Tumor Phenotype, Forming a Pro-Tumor Niche

Fibroblasts, as the main producers of collagenous fiber, participate in the formation and remodeling of extracellular matrix. Cancer-associated fibroblasts (CAF) are derived from fibroblasts and mesenchymal stem cells and have been shown to generate extracellular matrix components that contribute to the growth and progression of the tumor cells (59). Tumor-derived exosomes (or other extracellular vesicles) induce many types of cells to differentiate into CAF, thereby participating in extracellular matrix remodeling and reprogramming of the tumor microenvironment (17, 29, 60). Researchers have found that, in CLL, exosomes derived from tumor cells promote disease progression by transforming surrounding BMSCs into CAF, prompting proliferation of stromal cells and secretion of inflammatory cytokines of stromal cells, thereby promoting disease progression (51). Notably, exosomes derived from K562 cells, namely the CML cells line, containing human telomerase reverse transcriptase (hTERT) mRNA. These exosomes are transported into telomerase negative fibroblasts, then these mRNAs are translated into active enzymes in the recipient cell. Then these fibroblasts proliferate and live longer, becoming CAF that promote leukemia cells survival and disease progression (61, 62).

In AML, tumor-derived exosomes transfer multiple RNAs related to the pathogenesis of AML into mouse BMSC, including the mRNA of IGF-IR, CXCR4, and MMP9. The transfer of these transcripts promotes the proliferation of receptor cells and causes stromal cells to secrete growth factors that promote the growth of leukemia cells, leading to matrix remodeling (30, 63). Bone marrow stromal cells (BMSCs) treated with AML-derived exosomes increase the expression of genes that support the growth of tumor cells, such as IL-6 and CCL3, decrease the expression of genes that support normal hematopoiesis, such as CXCL12, KITL and IGF1 (31). Thus, through exosome-mediated information exchange, stromal cells increase their proliferation and survival, secrete more pro-leukemic cytokines, inhibit normal hematopoiesis, and promote disease progression.

Interleukin-8 (IL-8) belongs to the chemokine family, whose primary function is thought to be activation and chemotaxis of neutrophils. Corrado et al. found that, in chronic myelogenous leukemia (CML), tumor-derived exosomes containing amphiregulin (AREG), thereby activating epidermal growth factor receptor (EGFR) signaling in bone marrow stromal cells (BMSC), then the recipient stromal cells increase IL-8 expression. In addition, multiple myeloma-derived exosomes are also rich in AREG (64). Researchers also proved that IL-8 can promote leukemia cells survival *in vitro* and tumor growth of leukemia cells *in vivo* (39, 41).

Heparinase degrades heparin sulfate, a major component of the extracellular matrix, which results in extracellular matrix remodeling. In recent research, the role of heparinase in tumor progression has been gradually recognized (32). During therapy of myeloma, exosomes derived from tumor cells contain high levels of heparinase. Exposure of untreated tumor cells to these heparinase-positive exosomes activates ERK signaling, thereby regulating tumor cell survival. Besides, macrophages exposed to these heparinase-containing exosomes enhance their ability to secrete tumor necrosis factor-α (TNF-α), an important growth factor that promotes survival of myeloma cells (65).

### Other Mechanisms

Many tumor cells produce signature oncoproteins that play an important role in their own malignant proliferation. It has been found that tumor cells can package this signature oncoprotein (or oncoprotein-associated mRNA) into exosomes and deliver it to non-tumor cells, making it a more progressive phenotype and promoting the progression of tumor disease. Burkitt lymphoma cells are closely associated with Epstein-Barr virus (EBV) infection, and the infected tumor cells produce the latent membrane protein1 (LMP1) encoded by EBV. This protein mimics the CD40 signal and stimulates the growth, proliferation, and differentiation of B cells to a plasmacytoid phenotype, which is associated with an increased degree of malignancy. Researchers found that exosomes from Burkitt lymphoma cell lines contain LMP1, and primary B cells can bind and internalize these exosomes to induce cell proliferation and exhibit a malignant phenotype (66). In addition, the macrovesicles (exosome analogs) secreted by CML cells line K562 contain BCR-ABL1 mRNA, which can promote the transformation of bone marrow mesenchymal stem cells (BM-MSC) into a pro-tumor phenotype. These transformed cells can promote the proliferation of K562 cells and the expression of BCR-ABL (18).

## Exosomes Have the Function of Impressing Anti-Tumor Immune System

### Suppressing Anti-Tumor Immune Cells Directly

Under normal circumstances, natural killer cells (NK) and cytotoxic T lymphocytes (CTL) can recognize and kill malignant cells, and there are also artificially modified immune cells such as CAR-T and CAR-NK cells to kill tumor cells. However, tumor cells can evade the killing effect of immune cells through a variety of mechanisms to achieve the effect of immune evasion. Tumor cells secrete exosomes (sometimes only identified as small extracellular vesicles) containing a variety of immunosuppressive molecules, which can directly inhibit anti-tumor immune cells, such as NK, CTL cells (19, 42). For instance, exosomes obtained from plasma of acute myelogenous leukemia (AML) patients have been found to contain various immunosuppressive membrane molecules, including FasL, PD-L1, TGF-β (67).

For NK cells, activatory killer receptors that recognize non-MHC1 ligand molecules on their surfaces include NKG2D homodimer and natural cytotoxicity receptors (NCR). After NKG2D binds to its ligand, MHC class I chain-related molecules A/B (MICA/B), which expressed on the surface of target cells (namely tumor cells), NK cells can kill target cells and exert their immune surveillance function. Researchers have found that exosomes (or just small extracellular vesicles) derived from AML patients contain high levels of TGF-β1, which can down-regulate the expression level of NKG2D on NK cells, thereby inhibiting NK cell function (12, 68). Correspondingly, the number of NK cells and cytotoxicity in peripheral blood of AML patients are significantly reduced, as well as the expression level of NKG2D. Most importantly, the cytotoxicity of NK cells is restored after neutralization with the addition of anti-TGF-β1 antibodies (8). Interestingly, in T-and B cell leukemia/lymphoma, tumor cell increases MICA/B-containing exosome (or just small extracellular vesicles) secretion under oxidative stress, thereby preventing NKG2D from binding to the corresponding ligand on target cells by providing a decoy to bind NKG2D. This reduces the cytotoxic effect of NK cells on target cells, thereby promoting the immune escape of tumor cells (69).

Natural cytotoxicity receptors (NCR) include NKp46, NKp30 and NKp44, which can activate the killing effect of NK cells by transduction of activation signals (70). The ligand recognized by NCR hasn’t been thoroughly studied, but recent studies have found that BCL2-related athanogene-6 (BAG-6) is the ligand of NKp30 (71). Interestingly, binding of NKp30 with the soluble BAG-6 in plasma inactivates NK cells. In contrast, BAG-6-containing-exosomes can activate NK cells, enhance the cytotoxicity of NK cells, to better kill tumor cells. The level of BAG-6-containing exosomes in the plasma of patients with chronic lymphocytic leukemia (CLL) is decreased, and the level of soluble BAG6 is increased, which leads to the reduction of NK cells mediated killing of tumor cells. This suggests that the imbalance between BAG-6-containing exosomes and soluble BAG6 may mediate the immune escape of tumor cells (20).

For CTL, (namely CD8^+^T cells) they can express FasL, the ligand of death receptor Fas, to kill their target cells through the Fas/FasL pathway, and express the death receptor Fas to accept death signal. After CTL cells are activated and the immune effect is exerted, Fas on CTL cells binds with FasL on other immunosuppressive cells and mediates its own apoptosis, terminating the immune response in time, thus maintaining the immune balance of the body. However, tumor cells can also take advantage of this mechanism by secreting exosomes or other extracellular vesicles containing FasL to induce apoptosis of CTL cells, thus inhibiting the immune response to tumor cells (21, 72). Researchers have found that AML-derived exosomes mediate CD8^+^T cell apoptosis through Fas/FasL pathway, down-regulate the expression of CD3ζ, which is critical for transduction of activation signals, and down-regulate the expression of Janus kinase 3 (JAK3) in activated T cells. By these mechanisms, these exosomes inhibit the function of CD8^+^T cell (40). Besides, exosomes (or small extracellular vesicles) derived from tumor cells can inhibit CD8^+^T cells function through TGF-β (35).

### Activating Immunomodulatory Cells

CD4^+^CD25^+^Foxp3^+^T cells, also known as Treg cells, play an important role in immune regulation by suppressing the immune response through direct contact or by secreting inhibitory cytokines such as IL-10 and TGF-β. In patients with hematological malignancies, such as AML, the number of Tregs in peripheral blood and tumor tissues is increased (73). Researchers have found that exosomes in supernatant and serum of tumor cells from AML patients contain IL-10 and TGF-β1, and these exosomes promote CD4^+^CD25^-^ T cells proliferation and transformation into CD4^+^CD25^+^Foxp3^+^T cells. In addition, neutralizing antibodies specific to TGFβ1 and/or IL-10 inhibit the ability of these exosomes to amplify Treg cells (40).

MDSCs, namely Myeloid-derived suppressor cells, can produce immunosuppressive molecules to directly inhibit lymphocytes, and can also induce Treg production to indirectly inhibit immune response. It’s found that murine MM exosomes can regulate the signal transducer and activator of transcription 3 (STAT3), promote the growth of MDSCs and enhance its immunosuppressive activity *in vivo* (43).

M2-type macrophages can suppress the immune response by secreting inhibitory cytokines or direct contact, and participate in immune regulation. Plasma exosomes of CLL patients are rich in non-coding Y RNA hY4. Transfer of this exosome or hY4 alone to monocytes can facilitate their transformation into an immunosuppressive phenotype, namely M2-type macrophages. These transformed macrophages increase their expression of PD-L1 to inhibit immune cells. Besides, further studies show that hY4 transmits information through Toll-like receptors 7 (TLR7) (33). In addition, researchers found that diffuse large B-cell lymphoma-derived exosomes also promote the transformation of macrophages into the M2 phenotype (74).,

### Suppressing Anti-Tumor Immune Molecules

The immune system can rely on complement-dependent lysis to kill tumor cells. However, tumor-derived exosomes have been shown to contain casein kinase 2 (CK2), which phosphorylates C9 in the complement system and protects B-lymphoma cells from complement-mediated lysis. In addition, culturing Burkitt lymphoma cells with DRB (a selective inhibitor of protein kinase CK2) enhanced the killing effect of rituximab and complement for tumor cells (24). The complement treated K562 cells can package the membrane attack complex (MAC) inserted into their cell membrane surface into vesicles and release it out of the cell to protect themselves from killing. Mortalin has been shown to promote the secretion of MAC-laden vesicles in K562 cells (22). Whereas Mortalin inhibitor restore K562 cells sensitivity to complement-dependent cytotoxicity (75).

## Exosomes Have the Function of Favoring Therapy Resistance of Tumor Cells

### Exosomes or Exosome-Like Membrane Structures Can Sequester Intracellular Drugs or Excrete Them From Tumor Cells

Chemotherapeutic drugs can kill most tumor cells, but some tumor cells can use exosomes or similar membrane structures to evade killing through multiple mechanisms. For example, some tumor cells can actively enrich drugs in intracellular vesicles, and then secrete such membrane structure out of the cell, to maintain a low concentration of drugs in tumor cells. Or tumor cells may simply accumulate the drug in intracellular vesicles, isolating the drug from other components in the cytoplasm. Then the tumor cells acquire resistance to drug therapy.

In acute lymphoblastic leukemia (ALL), for example, p-glycoproteins have been found to be incorporated into exosome-like membrane structure, microparticles, and these transport proteins can promote the influx of anthracyclines in tumor cells into the microparticles, thereby trapping the drugs in such membranes. So, the chemotherapeutic drugs can’t kill the tumor cells, and the tumor cells develop therapy resistance (44). In addition, ABCA3 locates on the lysosome and intracellular multivesicular bodies in drug-resistant tumor cells in AML, realizing subcellular drug isolation and endows tumor cells with drug resistance (23, 25).

Diffuse large B-cell lymphoma cells treated with chemotherapeutic agents can expel the chemotherapeutic drugs doxorubicin and pixantrone from the cell by secreting drugs-containing exosomes. Treatment of lymphoma cells with the indomethacin can reduce expression levels of ABCA3, which is important for exosome biosynthesis, and thus inhibiting exosomes biogenesis. This causes doxorubicin and pixantrone drugs to accumulate longer in tumor cells and increases the sensitivity of tumors to these drugs (76).

In addition to these drugs, tumor cells may also excrete other endogenous substances that are not conducive to their survival by secreting extracellular vesicles (EVs). For example, the expression levels of miR-196 and miR-20a are higher in drug-sensitive AML cell line HL60, but lower in drug-resistant cells HL60/AR, and higher levels of miR-196 and miR-20a are found in small extracellular vesicle (sEV) secreted by drug-resistant cells. This selective expulsion of specific miRNAs from cells may be a way for drug-resistant cells to maintain drug resistance. However, this still needs further research (46).

### Exosomes and ATP-Binding Cassette Transporter

ABC transporters, namely ATP-binding cassette transporters (ABC), are transmembrane proteins which contains an ATP-binding cassette domain. They are encoded by a large gene family and is highly conserved in long-term evolution. Under normal physiological conditions, ABC protein exists on the plasma membrane and mediates transmembrane transport of sugars, amino acids, and peptides. A variety of drug-resistant tumor cells express ABC protein, which can transport anticancer drugs or endogenous anticancer molecules to the extracellular by means of energy generated by ATP hydrolysis, resulting in drug resistance of tumor cells. Recently, researchers found that exosomes mediate the horizontal transfer of drug resistance properties between tumor cells by transporting ABC-related molecules, such as proteins and mRNAs.

ABCB1 encodes p-glycoprotein (p-gp), namely multidrug resistance protein-1 (MDR-1). Drug-resistant ALL cells line VLB100 (high ABCB1 expression) can directly deliver functional P-gp protein to drug-sensitive ALL cells line CCRF-CEM *via* membrane-coated particles. CCRF-CEM cells integrate P-gp into their cell membrane and become a drug-resistant phenotype (47). In addition, exosomes from the multidrug-resistant osteosarcoma cell line MG63/DXR30 contain high levels of MDR-1 mRNA. After incubating these exosomes with parental MG-63 cells, MDR-1 mRNA and P-gp in parental MG-63 cells increase, and the sensitivity of parental MG-63 cells to adriamycin also decrease. This suggests that drug resistance can be transmitted between tumor cells not only through protein levels but also through mRNA levels (45).

ABCC1, namely multidrug resistance associated protein 1 (MRP1), is another ABC transporters associated with multidrug resistance. In acute promyelocytic leukemia (APL), APL cells line HL60/AR shows high expression of MRP-1, and they are highly resistant to daunorubicin. Researches treated drug-sensitive HL60 cells with extracellular vesicles produced by HL60/AR, and they found that HL60 cell line begin to express MRP-1, and increase resistance to daunorubicin. This establishes a transfer of drug resistance between the two strains (46, 77, 78).

### Exosomes That Carrying Decoy Can Reduce the Effectiveness of Monoclonal Antibodies

CD20 is expressed on the surface of B cells at all stages of development and differentiation except plasma cells, and plays an important regulatory role in the proliferation and differentiation of B cells. Anti-CD20 monoclonal antibodies such as rituximab can treat B-cell lymphoma and chronic lymphocytic leukemia by killing tumor cells through mechanisms such as antibody-dependent cytotoxicity, complement-dependent cytotoxicity, and inducing apoptosis. However, invasive B-cell lymphoma cells can release exosomes containing CD20 to combine with rituximab, reducing their bioavailability, depleting complement, and protecting target cells from antibody attack. Researchers have now discovered that exosome biogenesis is regulated by ATP-binding cassette protein A3 (ABCA3). Pharmacological blocking and/or silencing of ABCA3 inhibits exosome release, thereby enhancing the sensitivity of lymphoma cells to rituximab (79).

## Future Perspectives

So far, we have discussed the role of exosomes (sometimes small extracellular vesicles) in various hematological malignancies. And all that is known is for applications, and we have to talk about exosomes applications in oncology. The applications I see as promising fall into two categories. First, since tumor cells can promote the progression of disease through exosomes, we can use drugs to inhibit the synthesis of exosomes or other extracellular vesicles, so as to achieve the purpose of inhibiting tumors (80). Of course, in this process, attention should be paid to the problem that while inhibiting the synthesis and secretion of exosomes in tumor cells, drugs will definitely affect the synthesis of exosomes in normal cells. While using drugs to achieve this goal, we should maximize the impact on tumor cells while minimizing the impact on normal cells. This requires us to distinguish tumor exosomes from normal exosomes as much as possible, and it is best to enable drugs to target tumor exosomes. Second, with the current technical level, synthetic exosomes or other extracellular vesicles can be realized (81). We already know that there is a specific integrin profile on the surface of exosomes, which can enable exosomes to target and bind to corresponding cells. Can we consider synthetic exosomes, whose surface has specific membrane molecules that can target and bind to tumor cells. Besides various tumor suppressor molecules, such as cytotoxins, antibodies and cytokines, can be inserted into synthetic exosomes to achieve precise delivery of anticancer drugs. In addition, using exosomes to exert antitumor effect should have a broader prospect in hematological malignancies. Because exosomes are always transported through body fluids, and hematological malignancies contain most of their tumor cells in the blood, this makes it more likely that these methods will be used in hematological malignancies first.

## Conclusion

In some respects, tumor cells embody Darwinian evolution theory- not fit to die, fit to live. Tumor cells survive in their own microenvironment through a variety of mechanisms. For example, these tumor cells disable the immune cells that were supposed to kill them, making immune-regulatory cells more powerful. Not only do they secrete pro-survival factors that promote their own survival and inhibit apoptosis, but they also recruit otherwise neutral stromal cells to help them survive. They can reprogram their microenvironment to produce more blood vessels to supply them with nutrients and oxygen, and more pro-survival cytokines to help them survive, develop, and metastasize. When drugs are used to kill them, they can fight for survival through various mechanism. Tumor cells can use molecular pumps to flush drugs out of their cells, or sequester drugs in vesicles inside cells, reducing drugs impact on tumor cells themselves. They can even release decoys that bind to drugs and protect themselves from damage. Exosomes and other similar membrane structures can be involved in all these processes, on the one hand promoting tumor cell survival, on the other hand reducing the therapeutic effect. In this review, we introduce in detail the relevant studies on the role of exosomes in hematological malignancies in recent years, hoping to promote in-depth research in this area and conquer hematological malignancies as soon as possible.

## Author Contributions

HW made the figures; XZ and HW wrote the paper; XZ and YY reviewed the paper. All authors contributed to the article and approved the submitted version.

## Conflict of Interest

The authors declare that the research was conducted in the absence of any commercial or financial relationships that could be construed as a potential conflict of interest.

## Publisher’s Note

All claims expressed in this article are solely those of the authors and do not necessarily represent those of their affiliated organizations, or those of the publisher, the editors and the reviewers. Any product that may be evaluated in this article, or claim that may be made by its manufacturer, is not guaranteed or endorsed by the publisher.

## Abbreviations

AML, acute myelogenous leukemia; APL, acute promyelocytic leukemia; BM-MSC, bone marrow mesenchymal stem cells; BMSC, bone marrow stromal cell; CAF, cancer-associated fibroblast; CK2, casein kinase 2; CLL, chronic lymphocytic leukemia; CML, chronic myelogenous leukemia; EGFR, epidermal growth factor receptor; EVs, extracellular vesicles; FGF, fibroblast growth factor; FIH-1, factor-inhibiting hypoxia-inducible factor 1; HIF-1, hypoxia inducible factor-1; hTERT, human telomerase reverse transcriptase; HUVECs, human umbilical vein endothelial cells; IL-8, Interleukin-8; LMP1, latent membrane protein1; MAC, membrane attack complex; MDSC, myeloid-derived suppressor cell; MDR-1, multidrug resistance protein-1; MICA/B, MHC class I chain-related molecules A/B; MRP1, multidrug resistance associated protein 1; NCR, natural cytotoxicity receptors; P-gp, p-glycoprotein; sEV, small extracellular vesicles; STAT3, signal transducer and activator of transcription 3; TGF-β, transforming growth factor-β; VEGF, vascular endothelial growth factor.
